# Seasonal water-heat-salt dynamics in coastal salinized fields: impacts on cotton photosynthesis and yield

**DOI:** 10.3389/fpls.2025.1600293

**Published:** 2025-07-14

**Authors:** Guoyi Feng, Qian Zhang, Yan Wang, Ming Dong, Shulin Wang, Zhe Wu, Hong Qi

**Affiliations:** ^1^ Cotton Research Institute, Hebei Academy of Agricultural and Forestry Sciences, Shijiazhuang, China; ^2^ National Cotton Improvement Center/Key Laboratory of Cotton Biology and Genetic Breeding in Huanghuaihai Semiarid Area, Ministry of Agriculture and Rural Affairs, Shijiazhuang, China; ^3^ Institute of Coastal Agriculture, Hebei Academy of Agricultural and Forestry Sciences, Tangshan, China

**Keywords:** cotton cultivation, saline-alkali land, photosynthetic characteristics, water-heat-salt dynamics, soil salt-moisture movement

## Abstract

**Introduction:**

Soil moisture, temperature, and salinity critically constrain cotton production in saline soils. Understanding how cotton photosynthetic characteristics and yield respond to seasonal water-heat-salt dynamics is essential for improving management practices in these challenging environments.

**Methods:**

A two-year field study (2015–2016) was conducted comparing cotton growth, photosynthetic characteristics, and yield in mildly, moderately, and severely salinized fields. Seasonal soil water-salt dynamics in the 0–200 cm layer were monitored.

**Results:**

seasonal rainfall and temperature fluctuations significantly influenced soil water-salt dynamics in the 0-140 cm layer. During spring (April-May), drought maintained soil salinity above 3 g/kg, while low temperatures delayed cotton germination by over a day. In rainy season (July-August), rainfall leached salts from topsoil (0-40 cm), reducing salinity to below 5 g/kg and alleviating salt stress. Mildly saline fields exhibited superior photosynthetic performance, with leaf area index, chlorophyll content, and canopy photosynthetic rate being 1.2–1.5 times higher than in moderate/severe fields. These fields also showed extended "source-sink" organ development periods (16–28 days for "source," 4–24 days for "sink") and 13.8%–18.8% greater boll weight, ultimately achieving a seed cotton yield of 3,303 kg/ha—34.3%–120.7% higher than yields from moderate/severe fields.

**Discussion:**

Our results indicate that excessive soil salinity primarily impairs photosynthetic capacity and disrupts photosynthate allocation to bolls. Strategic interventions like drip irrigation could mitigate salt stress while improving photosynthetic efficiency and yield, providing practical solutions for cotton cultivation in saline fields.

## 1 Introduction

The utilization of saline-alkali soil has long been a critical concern in global agriculture, particularly in regions with limited arable land resources. China alone possesses nearly 100 million hectares of saline-alkali land, approximately 40% of which is located in coastal areas. These coastal saline-alkali soils, characterized by annual precipitation exceeding 500 mm, exhibit significant agricultural potential (Gang et al., 2024). However, soil salinization in these regions severely disrupts physicochemical properties, leading to constrained crop productivity (Rengasamy, 2010). Given China’s pressing need to ensure food security amid dwindling fertile land, the rational exploitation of saline-alkali soils is imperative.

Elevated soil salinity remains the primary constraint to agricultural productivity in these areas, as it directly influences water and salt dynamics through interactions with environmental factors such as temperature, rainfall, and wind. For instance, seasonal variations significantly modulate salt movement: intense evaporation during arid springs exacerbates surface salt accumulation, while summer rainfall facilitates downward leaching of salts from the root zone (Li et al., 2022). Such dynamics underscore the importance of understanding water-salt transport mechanisms in saline soil management (Rengasamy, 2010). Current reclamation strategies—including engineering, chemical, and biological approaches—have predominantly focused on mitigating soil salinity. Among these, biological methods, particularly the cultivation of salt-tolerant plants, have gained prominence due to their ecological sustainability (Xu et al., 2023).

Soil salinity adversely affects plant growth by inducing osmotic stress, ion toxicity, and oxidative damage, which in turn disrupts essential physiological and biochemical processes such as root development, secondary metabolite synthesis, and photosynthesis For example, studies have demonstrated that salt stress reduces root hydraulic conductivity, thereby limiting water uptake and biomass accumulation (Fahad et al., 2015). Simultaneously, salinity activates stress-responsive genes and signaling pathways, inducing protective enzymes and metabolites like proline and antioxidants (Joshi et al., 2022). Photosynthesis, a cornerstone of crop productivity, is particularly vulnerable to salinity; salt-induced stomatal closure, chlorophyll degradation, and photoinhibition collectively suppress photosynthetic efficiency (Chaves et al., 2009).

Cotton (*Gossypium hirsutum* L.), a globally significant cash crop, is renowned for its moderate salt tolerance and is often regarded as a pioneer species for reclaiming saline-alkali soils (Abdelraheem et al., 2019). Given that cotton fiber—a carbohydrate derived from photosynthetic products—represents its primary economic yield, it is essential to understand the photosynthetic responses to salinity (Zhang et al., 2017). Previous studies have established that salinity reduces net photosynthetic rates, alters chlorophyll fluorescence parameters, and diminishes leaf area index (LAI) in cotton (Huang et al., 2015). However, these investigations often overlook the dynamic interplay of annual variations in water, heat, and salinity, as well as their spatial-temporal heterogeneity in coastal saline-alkali ecosystems.

Notably, the coastal saline-alkali environment is characterized by typical fluctuations in soil moisture, temperature, and salt content across seasons, leading to complex stress interactions (Jia et al., 2024). Although isolated effects of salinity or drought on cotton physiology have been documented (Rehman et al., 2021), a holistic analysis of how multi-factor annual cycles influence photosynthetic performance and yield formation remains few. These hinder the development of targeted strategies for sustainable cotton cultivation in these marginal lands.

To address this, our study investigated the response of cotton’s photosynthetic characteristics and yield to annual variations in water, heat, and salinity across naturally saline-alkali soils in a coastal region. Over a two-year field experiment, we systematically monitored (a) seasonal dynamics of rainfall and temperature, (b) spatiotemporal shifts in soil moisture and salinity, and (c) their cumulative impacts on cotton growth stages. Key photosynthetic parameters—including canopy photosynthetic rate, respiration rate, chlorophyll content, LAI, and photoassimilate partitioning *etc.*—were analyzed to elucidate mechanistic linkages between environmental stressors and yield limitations. By integrating temporal and spatial dimensions of water-salt-heat interactions, this work aims to advance strategies for optimizing cotton productivity in saline-alkali agroecosystems.

## 2 Materials and methods

### 2.1 Description of the test site

The experiment was conducted from 2015 to 2016 at the state-owned Haixing Farm located in Hebei Province (latitude 38°21′ N, longitude 117°31′ E), which has consistently employed a monoculture system for cotton production. This region is located in the southeastern part of Hebei Province, on the western shore of Bohai Bay, and is characterized by a typical temperate monsoon climate with mean annual temperatures of 12–13°C and annual precipitation averaging 500–600 mm, predominantly concentrated in July and August (above 60% of total rainfall). The area receives about 2,600 annual sunshine hours, providing sufficient light for agricultural activities. The predominant soil types are gley soil and saline-alkali soil, with more severe salinization occurring along coastal areas. According to the salinity levels in the plow layer (0–20 cm depth) during the second half of April, three distinct zones were selected: mild, moderate, and severe saline-alkali regions; each zone covers an area exceeding three square kilometers. Detailed information regarding the salt content and nutrient composition of the plow layer soils across these three zones can be found in **Table 1**.

**Table 1:** Soil nutrient status of the cotton fields with differently salinized levels.

Salt content of plow-layer	Saline-alkali level	Organic matter	Total nitrogen	Alkali hydrolysablenitrogen	Available phosphorus	Available kalium
g/kg	-	g/kg	g/kg	mg/kg	mg/kg	mg/kg
5.0~10.0	Severe	7.67 ± 0.31^c^	0.44 ± 0.02^c^	20.06 ± 1.00^c^	3.88 ± 0.15^c^	215.54 ± 9.27^a^
3.0~5.0	Moderate	8.88 ± 0.44^b^	0.71 ± 0.03^b^	25.71 ± 1.26^b^	9.62 ± 0.38^b^	198.63 ± 9.31^b^
1.0~3.0	Mild	10.27 ± 0.50^a^	0.82 ± 0.03^a^	35.43 ± 1.47^a^	11.71 ± 0.48^a^	173.88 ± 6.69^c^

Different letters in the same column indicate a significant difference at the 0.05 probability level.

### 2.2 Cotton cultivation and management

**Figure 1:**
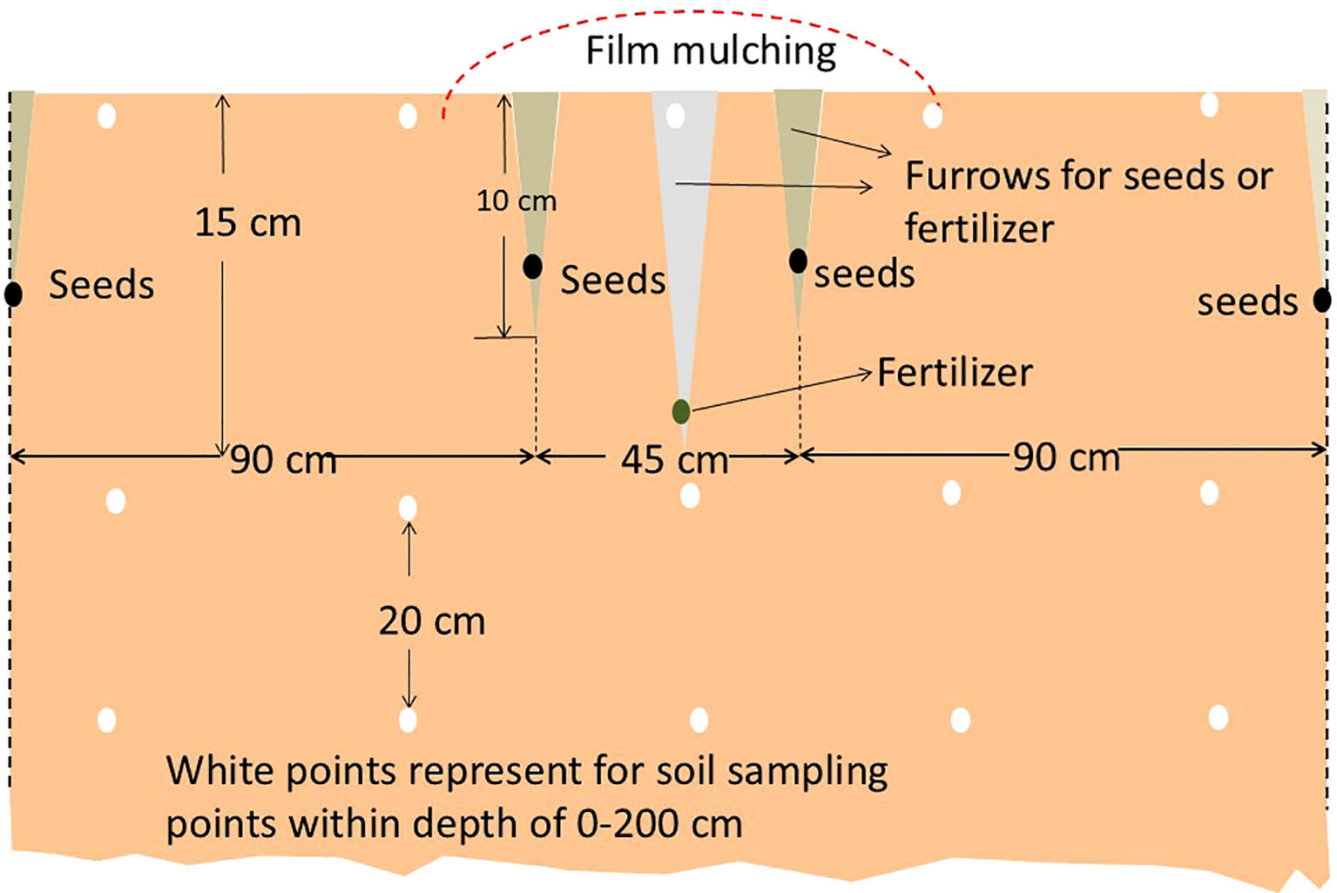
Cotton cultivation and design.

Cotton variety “Jimian 228,” jointly cultivated by the Cotton Research Institute, Hebei Academy of Agricultural and Forestry Sciences, and the Biotechnology Research Institute, Chinese Academy of Agricultural Sciences (Approval No. 2008003), was sown in April 2015 and April 2016, following rainfall. Cultivation followed local conventional practices, as illustrated in **Figure 1**. Pre-planting fertilization employed a split nitrogen (N) application strategy, wherein 60% of the total urea (270 kg N/ha) and superphosphate (750 kg/ha) were incorporated as basal fertilizers. The remaining 40% (180 kg N/ha) was applied as topdressing during the peak flowering-boll formation stage in July. This staged nitrogen management was designed to align with critical growth phases of cotton, thereby minimizing leaching and volatilization losses. Following fertilization, plastic film mulching was implemented. Topping was completed by July 20, accompanied by an additional urea topdressing of 150 kg/ha. This adjustment was made in real-time based on soil moisture levels and canopy nitrogen status, utilizing a SPAD-502 chlorophyll meter (SPAD-502, Minolta, Tokyo, Japan) with a threshold of SPAD set at ≤37.5 to ensure precision in nutritional management. Field management measures such as pruning, chemical control, and pest disease prevention were implemented according to conventional agricultural practices in the region.

### 2.3 Data collection

Cotton developmental stages were monitored post-sowing, including germination (field emergence rate ≥50%), seedling (coverage ≥50%), squaring (first flower bud visible in 50% plants), flowering (first flower opened in 50% plants), and boll opening (50% bolls split). Meanwhile, seasonal rainfall and temperature data during the cotton growing season (from April to October) were recorded. For soil analysis, six sampling points per field were established. Soil samples (0–200 cm depth, 20 cm intervals) were collected regularly on the middle of each month, homogenized by layer, and analyzed for moisture and salinity. Furthermore, various photosynthetic related indicators such as relative chlorophyll content (SPAD), leaf area index (LAI), canopy apparent photosynthesis rate (CAP), canopy respiration rate (CR), and dry matter accumulation and allocation were measured at key developmental stages—including squaring stage (after emergence around 60 days), flowering period (after emergence around 90 days), pre-boll opening stage (after emergence around 120 days), mid-boll opening stage (after emergence around 160 days), and post-boll opening stage (after emergence around 190 days). Finally, at harvest time for the cotton crop, yield measurements were taken. The specific measurement methods for the aforementioned indicators are detailed in the following section.

### 2.4 Methodologies for indicator detection

For the determination of soil moisture and salt contents, fresh soil samples were dried in an oven at 105°C for a duration of 6 hours firstly. The soil moisture content was assessed by measuring the weight of water lost relative to the dry soil mass, while soil salinity was calculated based on the weight of total soluble salts present in the soils compared to that of the dry soil. Detailed methodologies are described in reference (Zhang et al., 2017).

The Leaf Area Index (LAI) was directly measured using the LAI-2200 canopy analyzer (LI-COR, Lincoln, NE, USA) during early morning when there was no direct sunlight (Noor et al., 2023).

The canopy apparent photosynthesis (CAP) and canopy respiration rates (CR) were measured in the field using an infrared CO_2_ analyzer (GXH-305, Changchun Lepu Technology Co., Ltd.). Measurements were taken during stable light conditions of 1200~1400 μmol/m^2^/s on sunny days (9:00~11:00 Beijing time), with each measurement lasting 60 seconds. Simultaneously, soil respiration rates for each plot were recorded to calibrate CAP and CR values. For detailed descriptions, refer to literature (Noor et al., 2023; Noor et al., 2024).

To account for spatial heterogeneity and ensure representative sampling, three random points were systematically selected in each cotton field using a stratified grid approach. At each point, 10 fully expanded leaves from the upper canopy (4^th^ leaf of the main stem pre-topping; 2^nd^ leaf post-topping) were collected to balance statistical robustness and operational feasibility. SPAD values were measured *in situ* using a calibrated SPAD-502 chlorophyll meter (Minolta, Tokyo, Japan), following the manufacturer’s protocol. This methodology aligns with standardized practices for non-destructive chlorophyll estimation in field crops (Feng et al., 2014).

Dry matter accumulation and allocation. To ensure sample representativeness, six well-developed cotton plants were selected from each field, and were dissected into leaves, stems, buds, and bolls. These tissues were dried in an oven at 105°C for 30 min, then at 80°C until constant weight. A logistic model y=a/(1+be^−cx^) was used to simulate the dry matter accumulation process of “sources” (leaves + stems) and “sinks” (buds + bolls), where y represents weight of dry matter accumulation and x represents days after sowing (Feng et al., 2014).

Yield component quantification. At physiological maturity (prior to harvest in late October), six representative sampling plots (30 m² each) were established per field using a stratified random sampling design to capture field variability. Plot size and number were determined based on previous optimization trials to achieve a coefficient of variation <15% (Zhang et al., 2017). Plant density (plants/m²) and total boll count per plot were quantified to calculate boll retention rate (bolls/plant) and boll density (bolls/m²). Post-harvest, 50 intact bolls per plot were oven-dried at 65°C for 48 h to constant mass for determination of mean single-boll weight (g). Seed cotton yield (kg/ha) was computed by integrating boll density, boll weight, and harvest index (0.35). For full methodological details, refer to Zhang et al (Zhang et al., 2017).

### 2.5 Data analysis

Data was subjected to analysis of variance (one-way ANOVA) with the SPSS 16.0 software package (SPSS Inc., Chicago, IL, USA). Mean separation was analyzed using Duncan’s Multiple Range Test (DMRT) at p ≤ 0.05 or 0.01. All figures were created using Origin 8.0 software (OriginLab Corporation, North-ampton, MA, USA).

## 3 Results

### 3.1 Interannual temperature variations affect cotton growth stages in salinized fields

**Figure 2:**
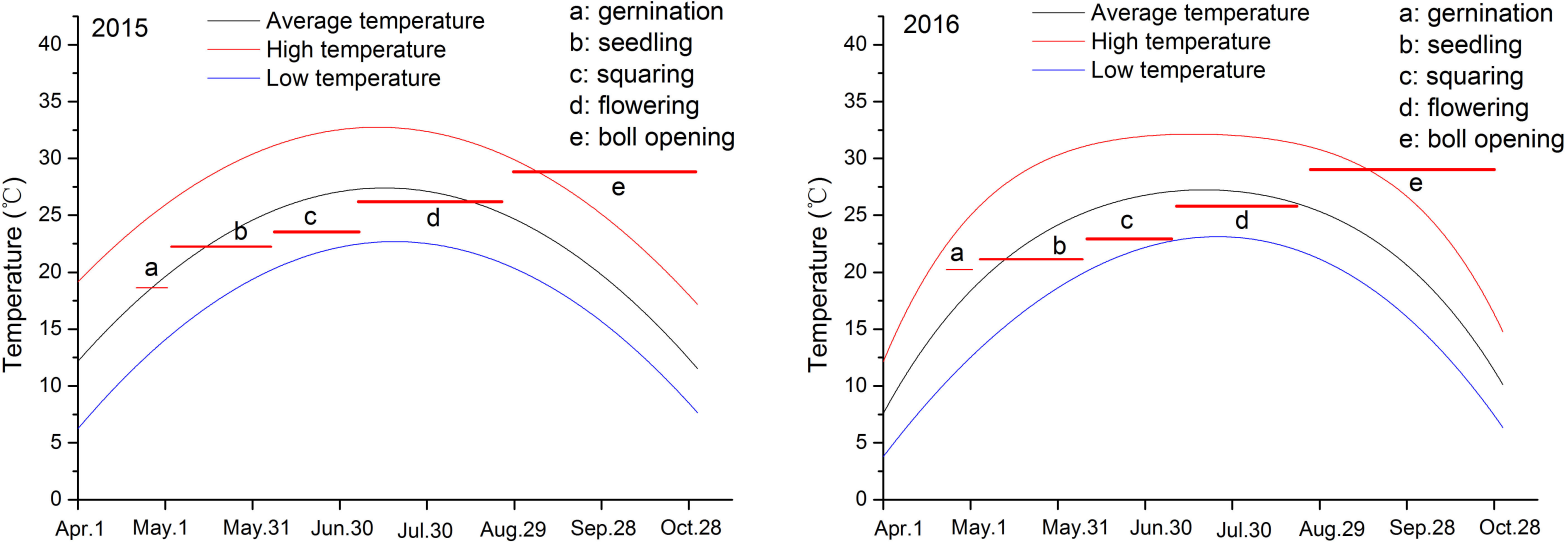
Temperature investigation for the year of 2015 and 2016 and cotton growth stages. Letters a-e represent different growth stages, and their linear lines represent duration and temperature.

Decreased temperature delayed cotton germination, while increased soil salinity promoted early maturation. In 2015 and 2016, the daily temperatures during the cotton growth period both ranged from 8 to 32°C, with an average of approximately 22.9°C, and showed the same interannual variation trend. However, from April to May, the average temperature in 2016 was slightly higher than in 2015. This was especially noticeable during the germination period, with an average temperature of about 20.9 °C in 2016, an increase of 2.7°C from 2015 (**Figure 2**; **Supplementary Table 1**); however, from May to June, it was somewhat lower. According to the data presented in **Table 2**, temperature influenced both germination and seedling stages: an increase in temperature shortened the germination time by one day, while a decrease extended the seedling stage by more than one day. Additionally, a reduction in soil salinity further prolonged the seedling stage (**Figure 2**; **Supplementary Table 1**). Moreover, increased soil salinity adversely affected seed germination and flower bud formation; under severely salinized fields, germination time was extended by over three days compared to mildly salinized fields. As soil salinity decreases, both flowering and boll opening periods were correspondingly prolonged (**Table 2**). Thus, it is evident that the synergistic changes in temperature and soil salinity influence cotton growth.

**Table 2:** The duration of cotton growth stages.

Salinized field	Germination stage ^A^		Seedling stage		Squaring stage		Flowering stage		Boll opening stage ^B^	
	2015	2016	2015	2016	2015	2016	2015	2016	2015	2016
Severe	10	10	38	38	27	26	48	47	70	71
Moderate	9	8	40	41	30	29	52	54	62	59
Mild	7	6	44	47	26	25	49	49	67	66

^A^Cotton was sown on April 21, 2015 and April 23, 2015, respectively; Values in the table represent the days after sowing; ^B^Cotton of the two years was harvested both on the end of October.

### 3.2 Rainfall and temporal and spatial variation of soil moisture

**Figure 3:**
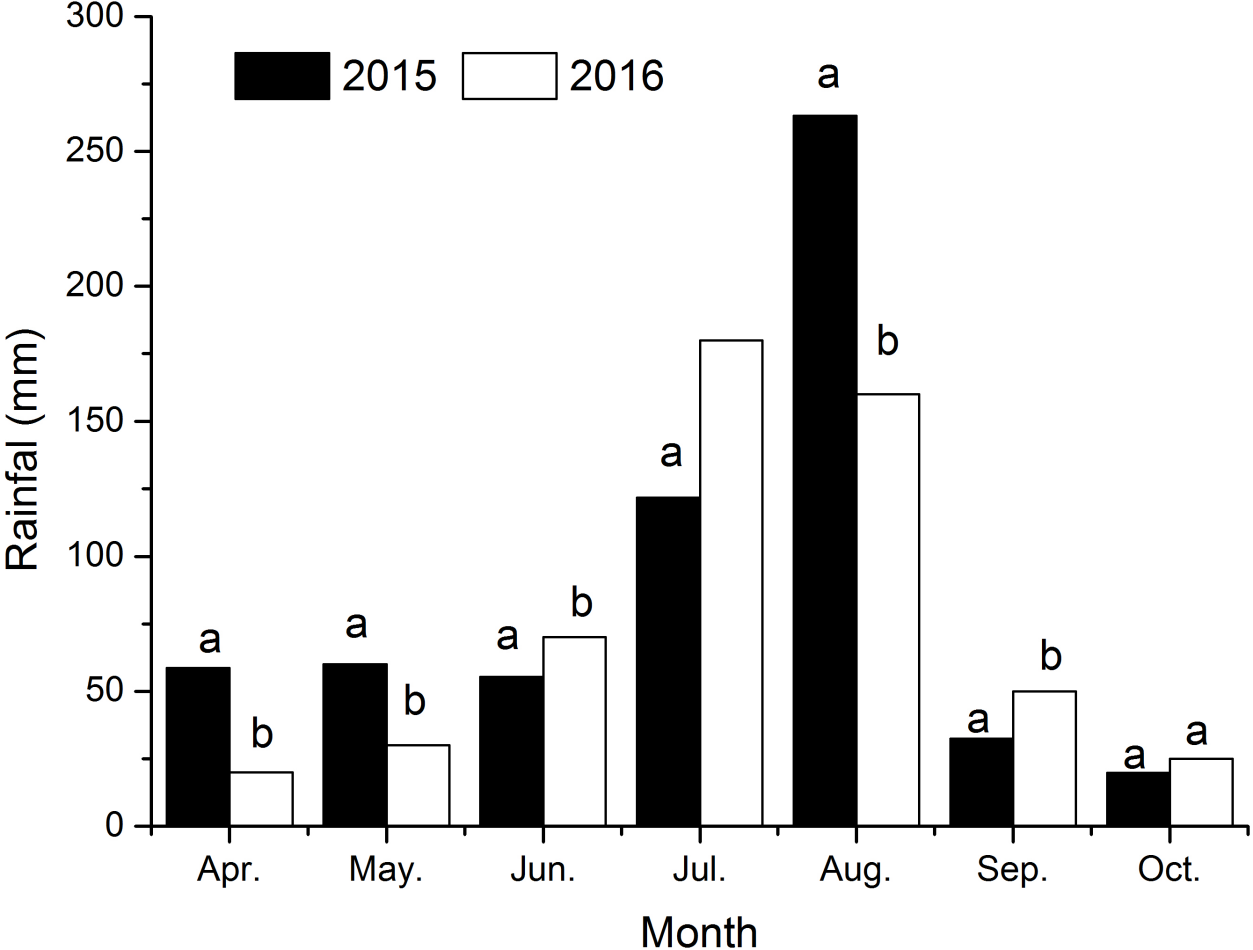
The sum of monthly precipitation in the year of 2015 and 2016. Different small letters in the same month indicate a significant difference between the two years at the 0.05 probability level.

**Figure 4:**
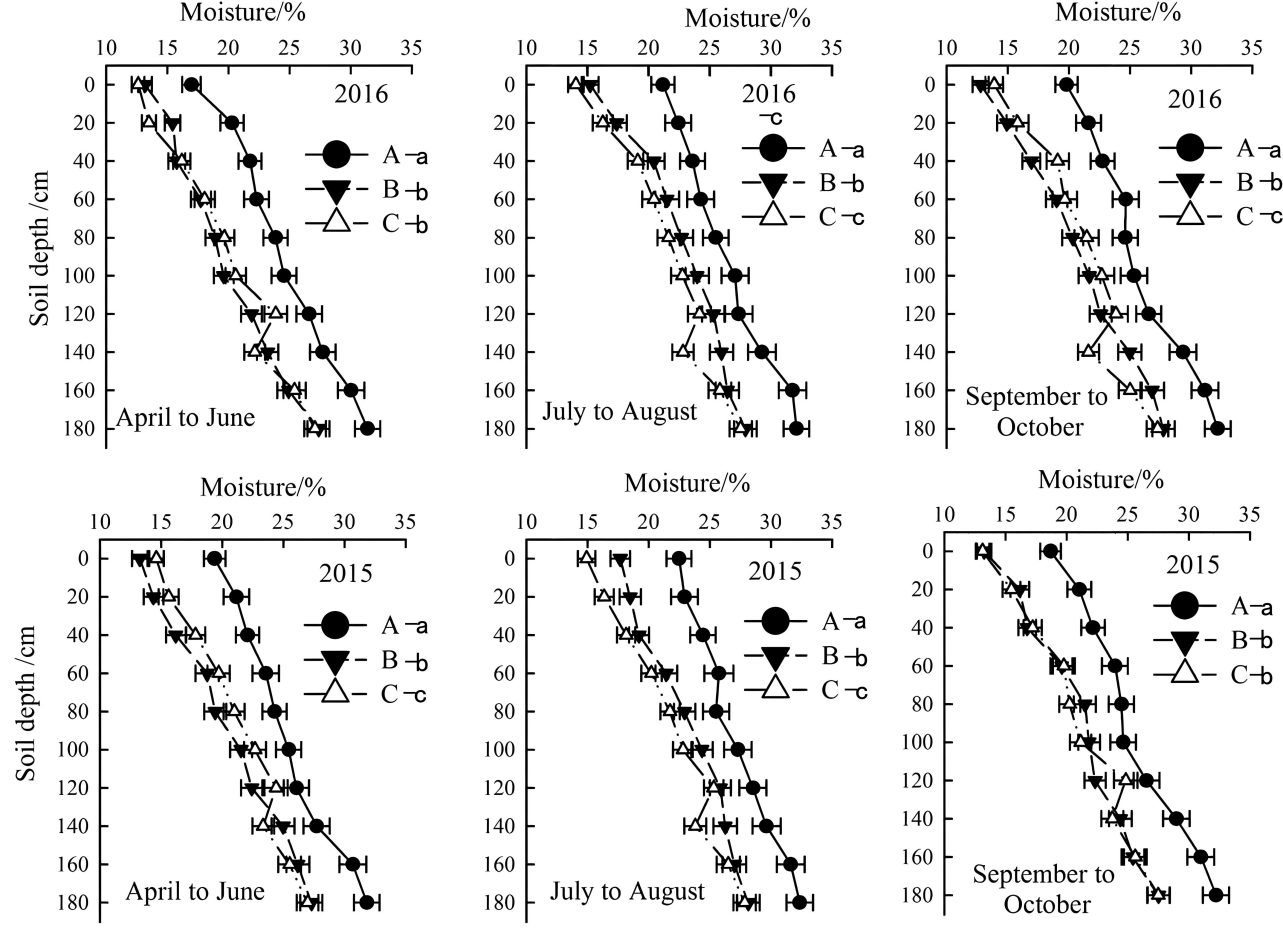
Temporal and spatial variation of soil moisture in the year of 2015 and 2016. Letter A, B and C represent severely, moderately and mildly salinized fields. Different small letters with A, B and C letters indicate a significant difference among the three fields within 0–140 cm layer at the 0.05 probability level. Data are mean ± SD, and Error bars indicates standard deviation.

The seasonal variation in rainfall directly affected soil moisture changes combining **Figures 3** and **4**. In 2015 and 2016, the total rainfall during the cotton growth period primarily concentrated in July and August, accounting for 63% and 63.5% of the total rainfall. The lowest rainfall occurred from April to June and September to October. Correspondingly, soil moisture in different soil layers varied obviously (**Figures 3**, **4**). Overall, soil moisture increased with depth, stabilizing below 140 cm, with the highest moisture content found in severely salinized soils.

From April to June, the soil moisture content in mildly salinized fields at a depth of 0–80 cm ranged from 12.6% to 19.8%, showing no significant difference compared to moderately salinized fields, but was lower by 11.4% to 30.5% than severely salinized fields. From July to August, the moisture content at a depth of 0–140 cm varied between 14.1% and 24.5%, which was lower by 4.6% to 15.5% than moderately salinized fields and by 9.6% to 33.7% than severely salinized ones. By September to October, the moisture content for this layer ranged from 13.9% to 24.4%, exceeding that of moderately salinized fields by 4.7% to 12.8%, yet still falling short of severely salinized fields by 4.2% to 29.8%.

### 3.3 Temporal and spatial variation of soil salt content

**Figure 5:**
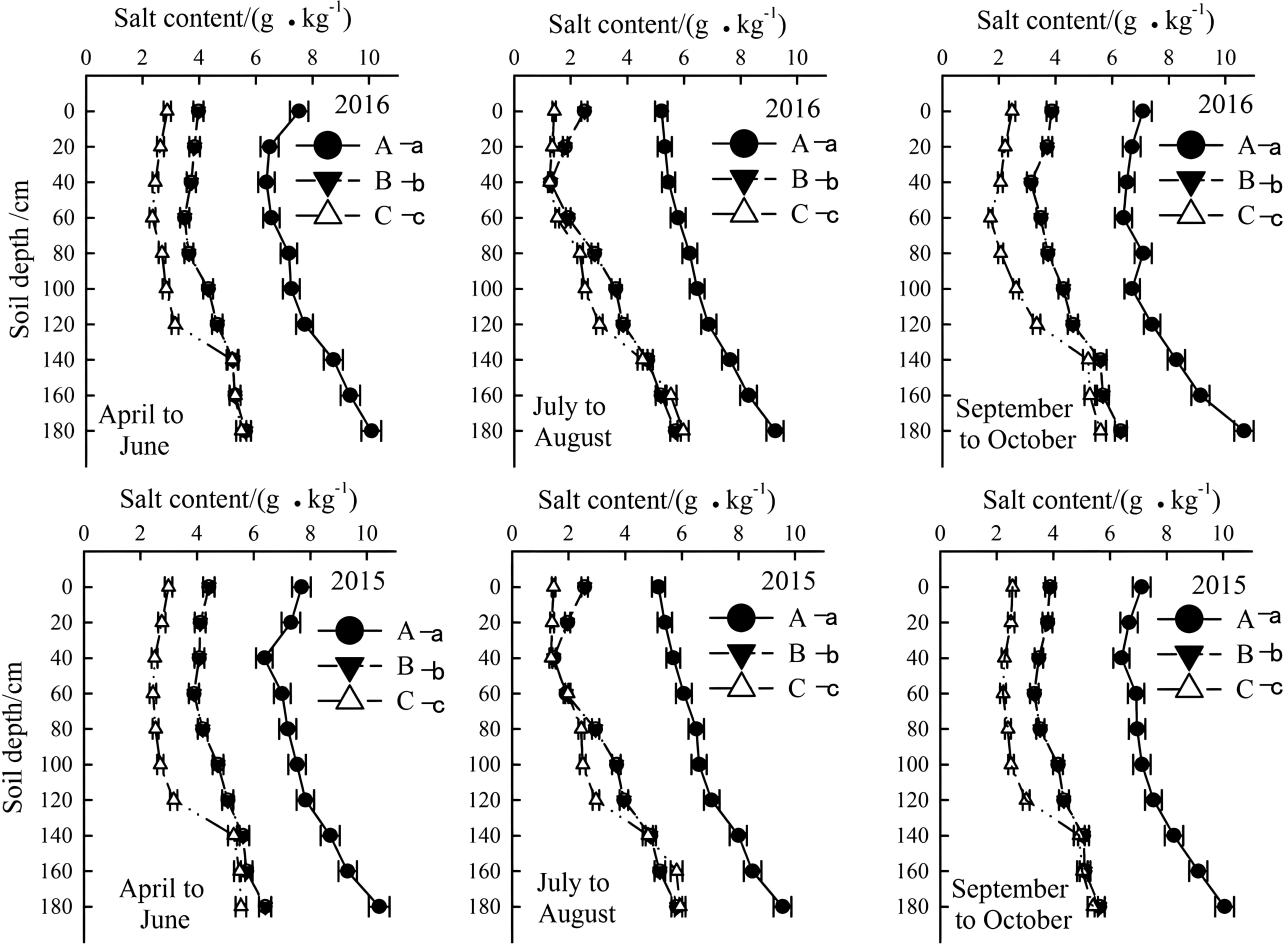
Temporal and spatial variation of soil salt content in the year of 2015 and 2016. Letter A, B and C represent severely, moderately and mildly salinized fields. Different small letters with A, B and C letters indicate a significant difference among the three fields within 0–140 cm layer at the 0.05 probability level. Data are mean ± SD, and Error bars indicates standard deviation.

The soil salinity in the 0–200 cm layer increased with depth and affected by seasons. In 2015 and 2016, the highest and lowest salinity levels occurred from April to June and July to August, respectively. Overall, the salt content in the 0–140 cm layer of mildly, moderately, and severely salinized fields ranged from below 3 g/kg to above 5 g/kg, while in the layer below 140 cm, it tended to stabilize. Nevertheless, the salt content in each soil layer of the severely salinized fields was obviously higher than that of the other fields (**Figure 5**).

Rainfall and soil moisture also influenced leaching of soil salts. From July to August, there was a significant reduction in salinity for all saline soils within topsoil layer (0–40 cm). Additionally, salt accumulation below 140 cm was significantly higher than in upper layers, indicating some degree of leaching.

### 3.4 Photosynthetic characteristics of cotton in differently salinized fields

#### 3.4.1 Leaf area index and chlorophyll content

**Figure 6:**
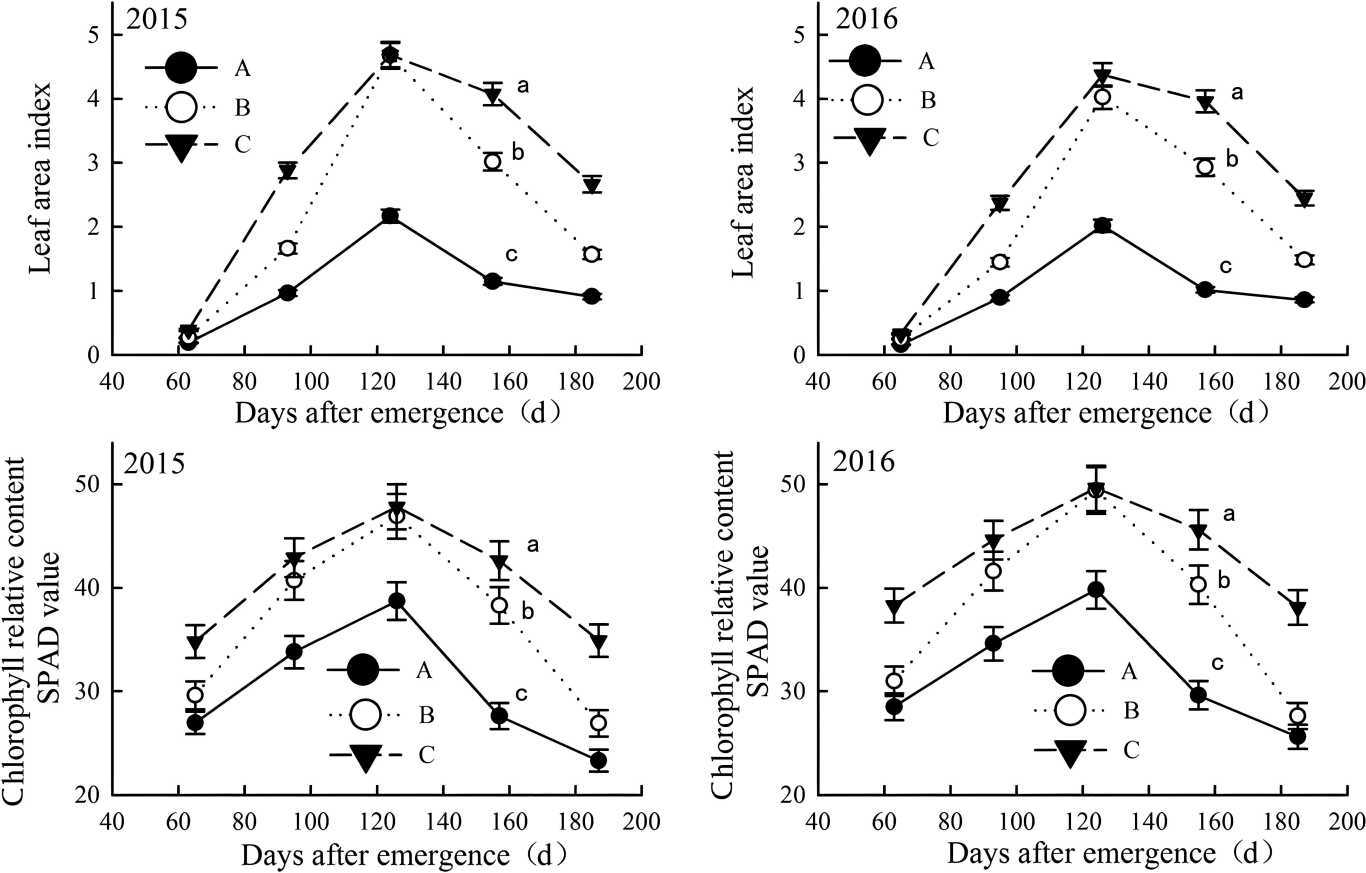
Leaf areas and relative chlorophyll content in salinized fields during the growth in the year of 2015 and 2016. Letter A, B and C represent severely, moderately and mildly salinized fields. Different small letters in A, B and C lines indicate a significant difference among the three fields during the whole growth period at the 0.05 probability level. Data are mean ± SD, and Error bars indicates standard deviation.

The leaf area index (LAI) and relative chlorophyll content of cotton increased with growth, peaking in late August (pre-boll opening stage) before declining; however, in more salinized cotton fields, these indicators were lower and declined more rapidly (**Figure 6**). In June and July (squaring stage and flowering period), these indicators were significantly lower in moderately salinized fields compared to mildly salinized ones. Prior to peaking in late August, the differences between the two indices gradually decreased and then narrowed rapidly. Mildly salinized cotton fields maintained higher values for both indicators, with a slower decline later on. The LAI ranged from 0.33 to 4.38, which was 105.3% to 290.0% higher than that of severely salinized fields, and 0.1% to 73.2% higher than moderately salinized ones. The SPAD value for relative chlorophyll content ranged from 34.8 to 49.6, exceeding those of severely and moderately salinized fields by 23.5% to 54.3%, and by 0.4% to 38%, respectively.

#### 3.4.2 Canopy apparent photosynthesis and canopy respiration rates

**Figure 7:**
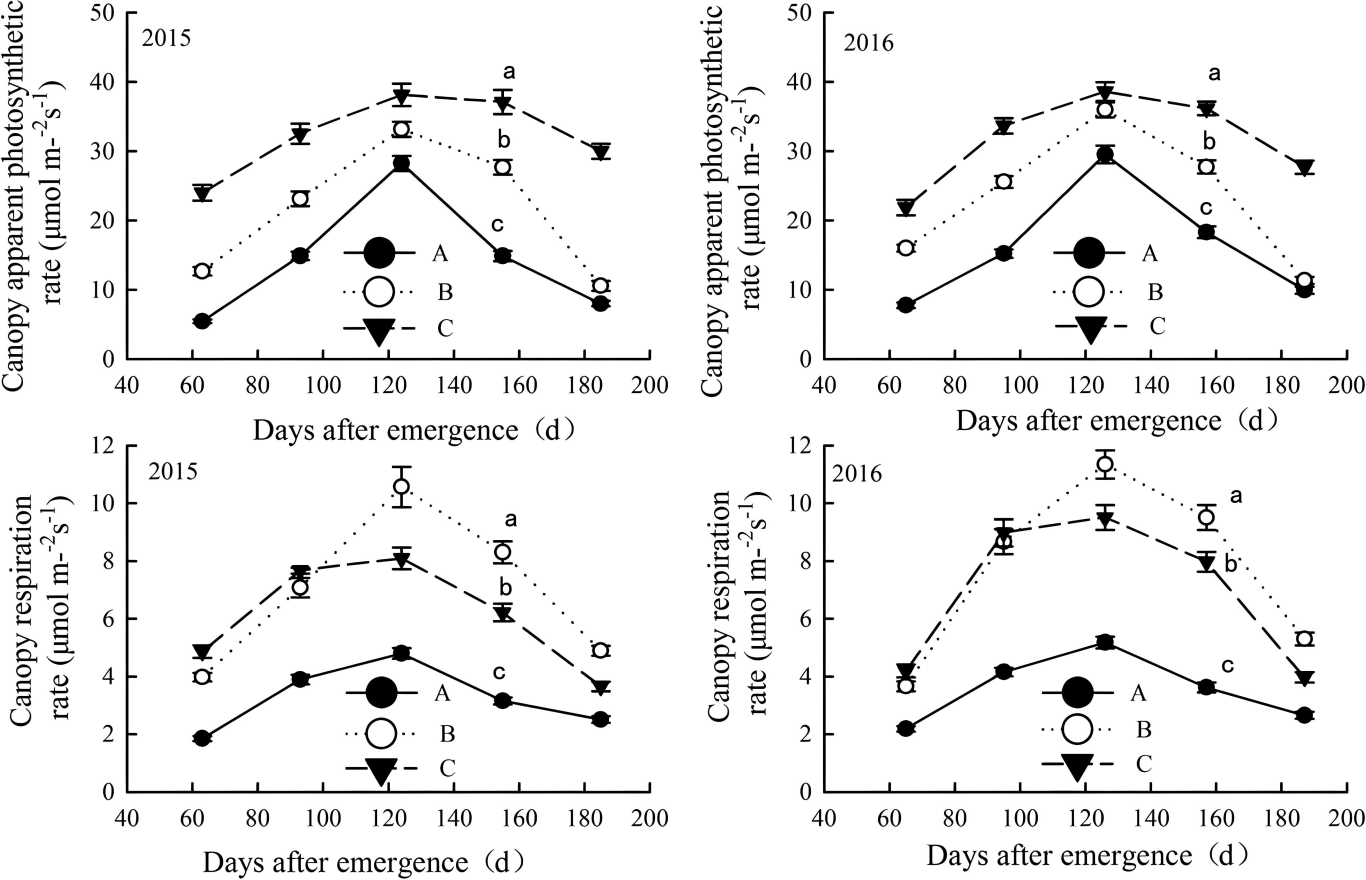
Canopy apparent photosynthesis and canopy respiration rates in salinized fields during the growth in the year of 2015 and 2016. Letter A, B and C represent severely, moderately and mildly salinized fields. Different small letters in A, B and C lines indicate a significant difference among the three fields during the whole growth period at the 0.05 probability level. Data are mean ± SD, and Error bars indicates standard deviation.

As the same pattern of LAI and relative chlorophyll content, CAP and CR of all salinized fields increased with cotton development, peaking in late August (pre-boll opening stage) before declining (**Figure 7**). The variations were significant across different salinization levels. severely salinized fields showed smaller values and fluctuations for the two indices, while mildly salinized cotton fields had higher values with a slower decline later on. In June and July (squaring stage and flowering period), the two indicators in moderately salinized fields were significantly lower than those in mildly salinized fields but approached or exceeded them after peaking in late August before rapidly decreasing.

The CAP of mildly salinized fields ranged from 21.8 to 38.6 μmol/m²/s, which was 30.9% to 339.5% higher than that of severely salinized fields and 7.4% to 183.6% higher than moderately salinized fields. The CR ranged from 3.65 to 9.51 μmol/m²/s, exceeding severely salinized fields by 46% to 162.8%, while being higher than moderately salinized fields during squaring stage and flowering period by 3.6% to 22.2%, but lower during the boll opening stage by 16.2% to 25.3%.

#### 3.4.3 Dry matter accumulation and allocation

In 2015 and 2016, the patterns of photosynthetic dry matter accumulation and distribution in sources and sinks were similar. Increased soil salinization led to reductions in total dry matter accumulation from photosynthesis (TPA), duration of linear growth (T_3_), maximum growth rate (*V_max_*) and the corresponding time (T_0_), as well as the active period of photosynthesizing substance accumulation (P). Additionally, salinization advanced both the start (T_1_) and end (T_2_) of the linear phase of dry matter accumulation (**Table 3**).

**Table 3:** Logistic equations for dry matter accumulation and allocation.

Salinized fields		Fitting equation	*R* values	TPA (kg/ha)	T_1_ (d)	T_2_ (d)	T_3_ (d)	*V*_max_ (kg/ha/d)	T_0_ (d)	P (d)
Source 2015	A	y=1 625.7/(1 + 4 971.2e^-0.1424x^)	0.9996**	1 645.0^c^	51^c^	69^b^	18^c^	48^c^	60^b^	42^c^
	B	y=3 833.0/(1 + 115.0e^-0.0606x^)	0.9938**	3 899.5^b^	58^a^	100^a^	42^b^	58^b^	78^a^	99^b^
	C	y=4 799.3/(1 + 75.6e^-0.0571x^)	0.9997**	4 822.8^a^	53^b^	99^a^	46^a^	69^a^	76^a^	105^a^
Source 2016	A	y=1 658.4/(1 + 368.2e^-0.1005x^)	0.9996**	1 675.9^c^	46^c^	72^b^	26^c^	42^c^	59^b^	60^c^
	B	y=3 904.7/(1 + 113.6e^-0.0621x^)	0.9953**	3 968.9^b^	55^a^	97^a^	42^b^	61^b^	76^a^	97^b^
	C	y=4 929.9/(1 + 55.5e^-0.0531x^)	0.9989**	4 971.7^a^	51^b^	100^a^	50^a^	65^a^	76^a^	113^a^
Sink 2015	A	y=674.8/(1 + 203.2e^-0.0789x^)	0.9905**	710.6^c^	51^c^	84^c^	33^c^	13^c^	67^c^	76^c^
	B	y=1 417.0/(1 + 252.9e^-0.0703x^)	0.9987**	1 439.8^b^	60^b^	97^b^	37^b^	25^b^	79^b^	85^b^
	C	y=2 746.5/(1 + 216.1e^-0.0546x^)	0.9980**	2 762.4^a^	74^a^	122^a^	48^a^	38^a^	98^a^	110^a^
Sink 2016	A	y=687.4/(1 + 160.4e^-0.0789x^)	0.9905**	723.9^c^	48^c^	81^c^	33^c^	14^c^	64^c^	76^c^
	B	y=1 434.7/(1 + 166.0e^-0.0665x^)	0.9961**	1 465.5^b^	57^b^	97^b^	40^b^	24^b^	77^b^	90^b^
	C	y=3 071.1/(1 + 120.2e^-0.0465x^)	0.9927**	3 092.8^a^	75^a^	131^a^	57^a^	36^a^	103^a^	129^a^

Source refers to the total dry weights of leaves and stems, while sink denotes the total dry weights of buds and bolls. Letter A, B and C represent severely, moderately and mildly salinized cotton fields. For the equation, y represents weight of dry matter accumulation and x represents days after sowing, and R value is the correlation coefficient of the fitting equation. ** indicates a significant difference at P<0.01 for R values. TPA, Vmax T_0_, T_1_, T_2_, T_3_ and P are derived from logistic equations. TPA represents total dry matter accumulation from photosynthesis; *V_max_* is the maximum growth rate. T_1_ and T_2_ indicate the start and end of the linear phase of dry matter accumulation (days after sowing). T_3_ denotes the duration (T_2_−T_1_), while T_0_ marks when the maximum growth rate occurs (days after sowing), and P signifies the active period of photosynthesizing substance accumulation (up to 90% of total accumulation). Different small letters signify significant differences at the 0.05 probability level within the same classification and year in a column.

For the source organs, the T_3_ period lasted from mid-June (squaring stage) to late July (late flowering stage), with the *V_max_* occurring in early June (beginning of squaring stage). Increased salt stress accelerated dry matter accumulation, while reduced salt stress prolonged this process. In mildly salinized fields, the T_1_ and T_2_ of the linear growth period and *V_max_* were delayed by 2–5 days, 28–30 days, and 16–17 days respectively compared to severely salinized fields. The T_3_, *V_max_*, and P in mildly salinized fields exceeded those in severely salinized fields by 89.2% to 98.7%, 57.1% to 71.3%, and 89.2% to 149.3%, respectively; they also surpassed moderately salinized fields by 6.0% to 16.9%, 8.0% to 18.1%, and 5.9% to 17.0%.

For the sink organs, the T_3_ period occurred from late June (late squaring stage) to late July (late flowering stage), with the *V_max_* reached in early July (beginning of flowering stage). Reducing salt stress benefited dry matter accumulation and significantly extended this process. In mildly salinized fields, the T_1_ and T_2_ of the linear growth period, as well as the *V_max_*, were delayed by 24–27 days, 38–50 days, and 31–39 days respectively compared to severely salinized fields. However, they occurred earlier by 14–18 days, 25–35 days, and 20–26 days compared to moderately salinized fields. The T_3_, *V_max_*, and P in mildly salinized fields exceeded those in severely salinized fields by 44.3%-69.6%, 163.4%-182.1%, and 44.3%-69.6% respectively; they also surpassed those in moderately salinized fields by 28.7%-43%, 49.7%-50.6%, and 28.7%-43%.

### 3.5 Cotton yield component analysis

In 2015 and 2016, the yields of seed cotton and lint cotton varied significantly across different salinized fields. As salinity increased, cotton yields decreased notably. In mildly salinized fields, the average seed cotton yield is 3303 kg/ha, with an average lint yield of 1274.9 kg/ha. This yield is 90.3% to 120.7% higher than in severely salinized fields and 34.3% to 38.5% higher than in moderately salinized fields. The average lint yield in moderately salinized fields is 921.6 kg/ha, exceeding that of severely salinized fields by 42.5% to 59.7%. Overall, yields in various salinized fields were slightly higher in 2015 compared to those in 2016 (**Table 4**). Further analysis revealed that mildly salinized fields exhibited significantly higher plant density, boll density, and boll weight compared to moderately and severely saline fields, with increases of 11.4%-44.8%, 10.9%-62.2%, and 13.8%-18.8%, respectively. Moreover, the differences in boll weight between severely and moderately salinized fields were not significant; however, it was significantly lower than that observed in mildly salinized fields. Therefore, the primary factors leading to reduced cotton yield in saline fields were attributed to boll density and boll weight from a “sink” perspective.

**Table 4:** Cotton yields assessment.

Year	Code	Plant density. (×10^4^ plants/ha)	Boll density (×10^4^ ·ha)	Boll weight (g)	Seed cotton yield (kg/ha)	Lint cotton yield (kg/ha)	Lint percentage (%)
2015	A	3.80 ± 0.17^c^	32.5 ± 1.53^c^	5.58 ± 0.20^b^	1 733 ± 48.9^c^	658.5 ± 21.3^c^	38.0 ± 1.86^b^
	B	4.57 ± 0.18^b^	47.5 ± 2.14^b^	5.51 ± 0.23^b^	2 456 ± 74.6^b^	938.2 ± 31.9^b^	38.2 ± 1.84^b^
	C	5.09 ± 0.22^a^	52.7 ± 2.48^a^	6.35 ± 0.28^a^	3 298 ± 115.4^a^	1 276.3 ± 42.8^a^	38.7 ± 1.91^a^
2016	A	3.48 ± 0.16^c^	28.8 ± 1.42^c^	5.36 ± 0.24^b^	1 499 ± 49.8^c^	566.6 ± 24.7^c^	37.8 ± 1.68^b^
	B	4.29 ± 0.18^b^	44.7 ± 2.15^b^	5.39 ± 0.21^b^	2 388 ± 66.6^b^	905.1 ± 22.7^b^	37.9 ± 1.74^b^
	C	5.04 ± 0.20^a^	52.8 ± 2.49^a^	6.37 ± 0.26^a^	3 308 ± 103.6^a^	1 273.5 ± 40.7^a^	38.5 ± 1.79^a^

Letter A, B and C represent severely, moderately and mildly salinized cotton fields. Different small letters in the same column indicate a significant difference at the 0.05 probability level.

## 4 Discussion

### 4.1 Comparison to prior studies of water-heat-salt interactions

The interplay of water, heat, and salt dynamics in coastal saline-alkali soils creates a complex environmental matrix that profoundly influences crop productivity, particularly in cotton (*Gossypium hirsutum* L.). This study demonstrates that soil salinization, driven by seasonal fluctuations in water and heat, directly impairs photosynthetic efficiency and yield formation in cotton. By systematically analyzing the temporal and spatial interactions of water-heat-salt dynamics, our findings corroborate and extend prior hypothesis that these interactions of water-heat-salt underpin soil salinization and its cascading effects on plant physiology (Abiala et al., 2018; Sun et al., 2018; Li et al., 2022). Below, we discussed our results within existing literature across three aspects.

#### 4.1.1 Water-heat-salt interactions and soil salinization

Our results align with global observations of seasonal salt migration in saline ecosystems but provide novel insights into coastal saline-alkali soils. The annual variations in rainfall and temperature significantly modulated soil moisture and salinity profiles (0–200 cm), as observed in the two-year field experiment. Summer rainfall facilitated downward salt leaching, alleviating root-zone salinity during critical growth stages (e.g., squaring and flowering), whereas spring droughts coupled with high temperatures exacerbated surface salt accumulation through enhanced evaporation (**Figures 4**, **5**). This pattern mirrors findings in arid regions, where water deficits amplify salt migration to upper soil layers (Rengasamy, 2006). However, coastal saline soils exhibit unique dynamics due to higher annual precipitation (>500 mm), which intensifies seasonal leaching-reaccumulation cycles (Rengasamy, 2006; Gang et al., 2024; Jia et al., 2024).

Notably, our study highlights synergistic temperature-salinity effects on germination. Research indicates that under suitable temperature conditions (25-30°C), cotton germination is typically delayed by 4 to 5 days (Reddy et al., 2017; Malima et al., 2024). Furthermore, studies have demonstrated that when soil salt content exceeds 3 g/kg, the germination time may be postponed by 5 to 15 days, and the germination rate declines to below 50% (Ashraf, 2002; Saboora et al., 2006). Here, a 2.7°C temperature increase in 2016 shortened germination by one day, even under elevated salinity, suggesting that warmer springs may partially offset salinity-induced delays—a phenomenon less explored in prior work. Additionally, as soil salinization increased, this accelerated germination effect became more pronounced. This reflects a synergistic effect between temperature and soil salinity. Furthermore, since this study was conducted with spring sowing after rain and the use of mulching measures, soil moisture did not have a significant impact on germination and the seedling stage. Therefore, it can be inferred that the combined effect of spring temperature and soil salinity jointly affected the germination and early seedling stage of cotton.

Summer rainfall’s role in salt leaching further underscores the significance of crop management practices in saline-affected regions. Current research indicates that when soil salinity exceeds 3g/kg, cotton root water absorption is impaired and growth restriction begins (Abdelraheem et al., 2019). However, research also reports that drip irrigation effectively leaches salt, with an optimal amount of 290 mm found for optimal yield, water use efficiency, soil desalination, and desalination efficiency (Sun et al., 2017; He et al., 2024). Our study found that topsoil (0–20 cm) salinity remained above 6 g/kg in spring and autumn, but dropped below 5 g/kg during the summer months of July and August, coinciding with an average rainfall of 361 mm. This decrease in soil salt content effectively alleviated salt stress. Particularly in moderately salinized fields, maintaining soil salinity below 3 g/kg supports the normal development of cotton (**Figures 3**, **4**). This aligns with the report that rainfall-driven leaching is a cost-effective strategy for coastal saline soils (Abdelraheem et al., 2019). However, our data reveal that even moderate salinity (3–5 g/kg) during critical growth stages limits cotton development, necessitating precision interventions beyond natural rainfall (Abdelraheem et al., 2019; Shokri et al., 2024).

#### 4.1.2 Salinity-induced constraints on photosynthesis

Soil salinity critically impaired cotton’s photosynthetic performance, as evidenced by reduced leaf area index (LAI), chlorophyll content (SPAD), and canopy apparent photosynthesis (CAP) in moderate-to-severe saline fields (**Figures 6**, **7**; **Table 3**). This result is consistent with global studies but with nuanced mechanistic insights. The accumulation of Na^+^ and compromised K^+^ homeostasis in cotton leaves under salinity likely induced stomatal limitation by disrupting guard cell osmoregulation, thereby restricting CO_2_ assimilation despite maintained chlorophyll content (Farquhar and Sharkey, 1982; Chaves et al., 2009). Simultaneously, elevated antioxidant enzyme activities (SOD, CAT *etc.*) under saline stress suggest a dual role of reactive oxygen species (ROS) scavenging in mitigating photodamage at PSII and ameliorating ionic toxicity through membrane stabilization (Feng et al., 2014; Fahad et al., 2015; Huang et al., 2015). These interconnected mechanisms—ion imbalance-driven stomatal limitation and reactive oxygen species-mediated photoinhibition—jointly affect the physiological responses of photosynthesis. In our study, severely saline soils reduced LAI by 105–290% compared to mild saline fields, exceeding the 25–50% declines reported in non-coastal systems (Feng et al., 2014; Zhang et al., 2017), likely due to compounded osmotic and ionic stress in dynamic coastal environments. However, the future research should deeply investigate leaf ion contents and antioxidant enzyme activities to clarify the physiological response pathways under salt stress.

Seasonal salinity fluctuations further modulate photosynthetic resilience. In summer (July-August), rapid increases in rainfall effectively reduce surface soil salinity below 5g/kg, leading to peak values in various photosynthetic indicators (**Figures 6**, **7**). This contrasts with arid regions, where salinity remains chronically high; however, temporary alleviation of stress can occur through leaching (Zhang et al., 2017). Temperature also plays a dual role: higher temperatures improved photosynthetic efficiency during leaching periods but exacerbated salinity stress during droughts (Singh et al., 2007). Such interactions emphasize the need for climate-adaptive management in coastal zones, but rarely quantified (Rengasamy, 2010).

#### 4.1.3 Dry matter partitioning and yield Implications

The source-sink imbalance under salinity stress, widely documented in crops, was starkly evident in cotton (Albacete et al., 2014). Cotton fibers, as the terminal storage form of carbohydrates, exhibit a developmental process that is highly dependent on the continuous supply and rational distribution of photosynthetic products. Research indicates that photosynthetic efficiency directly influences dry matter accumulation in cotton bolls and fiber quality (Feng et al., 2014). The dynamic balance between sources (leaves, stems) and sinks (cotton bolls, buds) is central to yield formation: when source organs provide sufficient carbon assimilates through photosynthesis, the development of sink organs can be optimized; conversely, salt stress disrupts this balance (Albacete et al., 2014). This study found that higher LAI, SPAD, and CAP in mildly salinized fields supported a prolonged linear accumulation period of dry matter (T_3_) and an increased the maximum growth rate (*V*_max_), thereby significantly enhancing yield (**Tables 3**, **4**). This finding aligns with the report that moderate salinity may enhance the utilization efficiency of photoassimilates (Chaves et al., 2009; He et al., 2022).

However, as salinity increases, the coordination between source and sink organs significantly decreases. In fields with severe salinization, salt stress leads to a reduction of 16–28 days in the linear accumulation period of dry matter (T_3_) for source organs and 4–24 days for sink organs. The *V*_max_ declines by 30%-50%, while the active period of photosynthetic product allocation (P) is reduced by 44%-69%. This inhibitory effect aligns with the mechanism that salinity disrupts phloem loading, resulting in a heightened sensitivity of reproductive organs to salt stress (Chaves et al., 2009; Baker and Baker, 2010). For instance, in severely saline fields, cotton boll density and weight decreased by 62.2% and 18.8%, respectively, compared to lightly saline fields, ultimately leading to a decline in seed cotton yield ranging from 93.8% to 124.8% (**Table 4**).

This study confirms that the synergistic fluctuations of water, heat, and salt in coastal saline-alkali soils indirectly influence the source-sink relationship of cotton by regulating soil salinity dynamics. Although the yield in mildly saline fields (3303 kg/ha) is significantly higher than that in moderately to severely saline fields, the leaching effect of summer rainfall— which reduces topsoil salinity in severely saline-alkali soils to below 5 g/kg—remains a critical period for alleviating salt stress. Future research should further elucidate the molecular mechanisms underlying the distribution of photosynthetic products in cotton under varying salinity gradients and explore synergistic strategies that combine precision irrigation (such as drip irrigation for leaching salts) with breeding for salt-tolerant varieties to optimize cotton productivity on marginally saline lands.

### 4.2 Adaptive strategies and management implications

The study highlights the potential of mild saline soils for sustainable cotton cultivation, as these conditions maintained higher photosynthetic efficiency and delayed senescence. Similar observations were reported by Hossain and Dietz, who noted that moderate salinity can trigger stress-acclimation mechanisms, such as osmolytes synthesis (e.g., proline) and antioxidant enzyme activation, to mitigate oxidative damage (Hossain and Dietz, 2016). However, in severe saline soils, such adaptations are insufficient to offset yield losses, necessitating integrated management strategies (Rengasamy, 2010; Hossain and Dietz, 2016). Our research indicated that in severely salinized fields, the plant density, boll density, and boll weight were significantly lower than in mildly salinized fields, with reductions ranging from 10.9% to 62.2%. These crucial indicators influence cotton yield. Therefore, in moderate or severe saline-alkaline conditions, implementing agricultural measures such as reducing soil salinity, improving seedling emergence rates, and increasing plant density can effectively ensure cotton production (Cao et al., 2024). For example, precision irrigation, tailored to seasonal rainfall patterns, could optimize salt leaching during critical growth stages (Sun et al., 2017; He et al., 2024). Furthermore, coupling soil amendments (e.g., biochar, gypsum) with water-saving irrigation may enhance soil structure and salt leaching efficiency, as demonstrated in recent trials (Shaygan and Baumgartl, 2022; Gang et al., 2024). Additionally, breeding for salt-tolerant cultivars with enhanced photosynthetic resilience—such as those with improved stomatal regulation or Na^+^ exclusion traits—could further mitigate salinity impacts (Parida and Das, 2005; Shaygan and Baumgartl, 2022).

## 5 Conclusions

This study, conducted over a two-year field experiment, elucidates the response of cotton photosynthetic characteristics and yield to the dynamics of water-heat-salt. The synergistic effects of water-heat-salt influenced the soil salinization process, which in turn had a chain reaction on cotton performance. The main conclusions are as follows:

### 5.1 Seasonal water-heat-salt dynamics critically govern soil salinization and cotton productivity in coastal saline-alkali ecosystems.

Rainfall and temperature variations directly modulated soil moisture and salinity profiles (0–200 cm), with summer precipitation (July–August) facilitating salt leaching in the root zone (0–140 cm). This seasonal desalination alleviated osmotic stress during critical growth stages (squaring and flowering), highlighting the importance of aligning irrigation practices with natural rainfall patterns to optimize salt management.

### 5.2 Mild saline-alkali conditions (soil salinity <3 g/kg) sustain superior photosynthetic performance and yield.

Under mild salinity, cotton exhibited enhanced leaf area index (LAI: 0.33–4.38), chlorophyll content (SPAD: 34.8–49.6), and canopy photosynthetic rates (CAP: 21.8–38.6 μmol/m²/s), which prolonged the linear accumulation period of photosynthetic dry matter (TPA: 4,822.8–4,971.7 kg/ha) and improved sink organ development. This resulted in a 34.3%–120.7% higher seed cotton yield (3,303 kg/ha) compared to moderate or severe saline fields, underscoring the viability of mild saline soils for sustainable cultivation.

### 5.3 Excessive salinity disrupts source-sink coordination, severely limiting yield potential.

Severe salinization (soil salinity >5 g/kg) reduced boll density by 62.2%, boll weight by 18.8%, and shortened the active photosynthetic period (47–58 days for source organs,11–44 days for sink organs). The yields of seed cotton and lint cotton in severely salinized fields were 33.3% to 51.1% lower than in moderately and severely salinized fields, primarily due to reduced boll density and weight. These effects stemmed from salt-induced inhibition of photosynthesis and impaired translocation of assimilates to reproductive organs, emphasizing salinity as a primary yield-limiting factor in coastal agroecosystems.

The increase of rainfall in summer promoted salt-leaching, but soil salinity in severely salinized fields was still above 5 g/kg, which limited cotton production. Thus, integrated management strategies are still essential for cotton production in severely salinized fields. This study advocates precision irrigation (e.g., drip systems) to synchronize salt leaching with summer rainfall, coupled with salt-tolerant cultivar breeding. Future works should prioritize field trials that combine soil amendments (such as biochar and gypsum) with water-saving technologies to enhance the cotton production in saline fields.

## Data Availability

The original contributions presented in the study are included in the article/**Supplementary Material**. Further inquiries can be directed to the corresponding author.
